# Postoperative Myocardial Infarction after Non-Cardiac Surgery: An Update

**DOI:** 10.3390/jcm13051473

**Published:** 2024-03-04

**Authors:** Carlo Rostagno, Anna Craighero

**Affiliations:** Department of Experimental and Clinical Medicine, University of Florence, 50121 Firenze, Italy

**Keywords:** non-cardiac surgery, myocardial infarction/injury, troponin

## Abstract

Every year, not less than 300 million non-cardiac surgery interventions are performed in the world. Perioperative mortality after non-cardiac surgery is estimated at 2% in patients over 45 years of age. Cardiovascular events account for half of these deaths, and most are due to perioperative myocardial infarction (MINS). The diagnosis of postoperative myocardial infarction, before the introduction of cardiac biomarkers, was based on symptoms and electrocardiographic changes and its incidence was largely underestimated. The incidence of MINS when a standard troponin assay is used ranges between 8 and 19% but increases to 20–30% with high-sensitivity troponin assays. Higher troponin values suggesting myocardial injury, both with or without a definite diagnosis of myocardial infarction, are associated with an increase in 30-day and 1-year mortality. Diagnostic and therapeutic strategies are reported.

## 1. Introduction

In patients undergoing major non-cardiac surgery, perioperative myocardial infarction (PMI) is a threatened complication. Before the introduction of biomarkers of ischemic damage and of troponin, postoperative myocardial infarction was diagnosed based on symptoms and electrocardiographic changes, and its incidence was largely underestimated. Analgesia often masks pain. Moreover, postoperative ECG monitoring is uncommonly utilized; therefore, transient ischemic changes may remain undetected, contributing significantly to a missed diagnosis. Myocardial damage that occurs after non-cardiac surgery is defined by the acronym MINS (Myocardial Injury after Non-cardiac Surgery). Myocardial injury, according to the IV universal definition of myocardial infarction, is defined by at least one troponin value above the 99th percentile of the upper reference values [1]. It is considered acute when associated with an increase/decrease in troponin values.

The diagnosis of postoperative myocardial infarction requires additional factors such as chest pain or other symptoms due to myocardial ischemia, new electrocardiographic ischemic abnormalities, development of pathological Q waves, evidence, with a diagnostic imaging technique, of a new loss of viable myocardium or new abnormalities of regional wall motion compatible with ischemic etiology, and, finally, evidence of a coronary thrombus by angiography or autopsy. 

In a small proportion of patients (<15 to 20 percent), perioperative myocardial injury has nonischemic causes (e.g., sepsis, tachyarrhythmias, heart failure).

Even with a clearer definition of the diagnosis of MINS and postoperative myocardial infarction, the relative difference in incidence between the two conditions may be conditioned by analgesic drugs that may mask symptoms, the absence of ECG monitoring, and/or the missing, despite troponin changes, of ischemic abnormalities when the ECG is repeated at fixed intervals after surgery. Moreover, the involvement of cardiologists in postoperative care is uncommon, leading to a lower sensitivity for acute cardiac complications. The clinical relevance of diagnosis of perioperative myocardial injury/infarction is sustained by survival data that report a significant increase in both early and 1-year mortality associated with increasing postoperative troponin levels. Moreover, the hospitalization rate for cardiovascular causes is significantly higher in comparison to patients without postoperative myocardial injury. Preoperative evaluation is often not fully exhaustive in risk stratification before surgery. In the perioperative period, routine evaluation of cardiac biomarkers is still largely underused, and the diagnosis underestimated. Finally, no clear indications have been provided about postoperative clinical evaluation, the need for coronary angiography (or ischemia imaging test), and, eventually, the treatment to limit late mortality.

## 2. Epidemiology

Every year in the world, about 300 million people undergo non-cardiac surgical procedures. Perioperative mortality is close to 2% in patients aged more than 45 years [2]. Cardiovascular events account for 50% of postoperative deaths [3]. About 170,000 cardiac complications related to non-cardiac surgery are reported in the US each year, with a 5% mortality [4]. Guidelines recommend performing a careful assessment of cardiovascular risk in patients undergoing non-cardiac surgery when high-risk procedures are scheduled, and postoperative troponin and NT pro-BNP monitoring [5].

In a large cohort study of patients aged >45 years, 8% had a cardiac event after non-cardiac surgery. More than 50%, however, did not meet the universal definition of MI [2]. Most of these events affect patients already suffering from coronary artery disease. The risk of MACE was particularly high within the first 7 postoperative days. This has a relevant clinical implication in considering therapeutic/preventive interventions aimed at preventing cardiac events.

In the first VISION study, 15,065 non-cardiac surgery patients aged >45 years had a measurement of troponin T in the first 3 postoperative days [6]. The incidence of MINS was 8%. Ischemic electrocardiographic findings (mainly T-wave inversion and ST depression) were found in less than 40% and only 41.8% would have fulfilled the universal definition of myocardial infarction. Vascular surgery (24.0%) was more frequently associated with myocardial injury. Myocardial injury was significantly associated with 30-day mortality (HR 2.2, 95% CI 1.9–2.6) and was one of the main causes of death. The use of a standard cTnT assay may have underestimated the true prevalence.

In the POISE study, in which a standard troponin assay (or alternatively CK-MB) was used for diagnosis, the incidence of postoperative MI was 5 percent at 30 days (4.2 and 5.7 in the beta blocker and placebo groups) [7]. Most (74%) occurred within 48 h of surgery. Also, in this study, symptoms occurred only in 35%. To be remarked upon, the finding of 30-day mortality was not different between asymptomatic MIs and symptomatic MIs (adjusted odds ratio 4; 95% CI 2.65–6.06 vs. 4.76; 95% CI 2.68–8.43, respectively). The non-uniform cut-off troponin in different centers, however, limits results interpretation.

A postoperative peak cTroponin I level ≥0.5 μg/L was found in 129/1030 elderly patients who underwent hip fracture surgery. In-hospital and 1-year mortality was significantly higher in patients with high troponin levels (12.5% vs. 3.5%, *p* = 0.0012 and, respectively, 44% vs. 16.1% at 12 months, *p* = 0.001 [8]. Coronary angiography was performed within 1 week of hip surgery in 18 patients. Multivessel coronary artery disease was found in all patients. One patient died after angiography. At multivariate logistic analysis, coronary revascularization (OR = 0.15, 95% CI = 0.03 to 0.78, *p* = 0.024) was an independent factor associated with improved survival while age and creatinine clearance were independent predictive factors of 1-year mortality. 

The BASEL-PMI study, a prospective single-center cohort study, evaluated high-risk patients defined as patients aged between 65 and 85 years or between 45 and 65 years and a history of cardiovascular disease (stroke, coronary artery disease, peripheral artery disease) undergoing non-cardiac surgery [9]. A total of 2265 patients, 43% of whom were female, underwent various non-cardiac surgeries (emergency, orthopedic, urological, thoracic, vascular, visceral) and were followed for one year. End points of the study were heart failure, clinically relevant arrhythmias, sudden cardiac death, pulmonary embolism, cardiovascular hemorrhage, and PMI (defined as the absolute hs-cTnT increase of 14 ng/mL above the preoperative concentration or between two postoperative determinations). To distinguish a PMI from a pre-existing hs-cTnT elevation, a preoperative measurement of hs-cTnT was used as a baseline. Two postoperative measures were obtained at the first and second day after surgery. The 365-day follow-up, completed in 99.5% of patients, showed an incidence of major cardiovascular events in 466 patients (20.6%), and the incidence of PMI alone was 14.8%.

Hs-cTnT was measured during the first three postoperative days in the second VI-SION cohort including 21,842 patients ≥45 years [10]. The diagnosis of MINS required an elevated postoperative hs-cTnT 20 to <65 ng/L with an absolute change of at least 5 ng/L between measurements or a single hs-cTnT level ≥65 ng/L. The perioperative myocardial injury was detected in 20%. Perioperative myocardial infarction was detected only in 846 patients (3.9 percent). A total of 93% of MINS and 68% of myocardial infarctions did not experience an ischemic symptom.

In high-risk populations, e.g., patients undergoing revascularization for critical limb ischemia, 25,5% had a myocardial injury (defined as hsTnT levels above the 99th URL of 14 ng/L and relative increase by ≥30% from the baseline level) [11]. One-year mortality was 14.2% and MACE incidence was 20.5%. Myocardial injury was an independent predictor of 1-year mortality and the risk of MACE increased from 3- to 5-folds in relation to hs-TnT levels. Again, in most patients with myocardial injury (85.2%), ischemic clinical symptoms or electrocardiography changes were not found.

Resuming the data from previously reported studies, the incidence of PMI varies from 3.5% to more than 20%. The demographic and therefore clinical characteristics of the population considered the relative risk related to each different site and type of intervention, the design of the study, the definition used for the diagnosis of myocardial infarction, and, finally, the troponin assay used to account for this large difference reported [12,13]. The introduction of the high-sensitivity troponin assay in fact led to a significant increase in MINS detection, from 8 to 19%, when standard cTnI-cTnT levels were assayed to the actual 20–30%. As will be discussed later, the clinical relevance of the introduction of the hs troponin assay in this setting should still be fully clarified.

## 3. Risk Factors

Accurate assessment of cardiac risk is critical before elective surgery to identify patients at high risk of PMI.

The main risk factors are divided into the following:

Preoperative risk factors: Age (>75 years) and comorbidities are the main risk factors in patients undergoing non-cardiac surgery. Although perioperative mortality from MI is higher in older people, age is not an independent predictor of perioperative cardiovascular risk. Main comorbidities include CAD, heart failure, hypertension, stroke, kidney failure, and diabetes. A known coronary artery disease is associated with a higher risk of postoperative myocardial injury and related mortality. The CARP trial [13] included patients with CAD with an indication of vascular surgery. MI was more frequent in patients aged >70 years, needing AAA surgery, symptomatic for angina, or with ECG ST-T abnormalities. In a subproject of the VISION study, 955 patients awaiting non-cardiac surgery underwent a preoperative coronary CT scan. Ninety-six percent of the 71 patients who developed PMI following surgery had extensive coronary artery disease on the coronary CT. Other factors related to an increased incidence of complications are emergency/urgent surgery and the patient’s nutritional and functional status. A compromised functional status correlates with a worse postoperative outcome: ADL and IADL are considered independent risk factors.

Intraoperative risk factors: Hemodynamic abnormalities in the operating room are associated with a higher risk of postoperative MI. The duration of intraoperative hypotension (mean arterial pressure < 55 mmHg) is an independent risk factor for PMI. Bradycardia, heart rate < 55/min, as well prolonged tachycardia, and heart rate > 110 are associated with a higher risk of MIMS [14,15]. Other factors include open surgery and the need for transfusions.

Postoperative risk factors: Postoperative bleeding, sepsis, hypoxia, sustained tachycardia, hypotension, and severe anemia are all factors associated with the risk of MINS. For every 1 g/dL Hb decrease after surgery, the risk of PMI increases by 1.46-fold [15].

Physicians have different tools to assess cardiac risk before surgery; however, validated risk models have been created. The 2016 guidelines of the Canadian Cardiovascular Society recommend risk stratification using the RCRI (Revised Cardiac Risk Index), a risk calculator for people over 45 years of age [16]. Only six variables are required for the risk to be quantified: a high-risk type of surgery including intrathoracic surgery, intraperitoneal surgery, and supra femoral vascular procedures, the presence of ischemic heart disease, the presence of congestive heart failure, cerebrovascular disorders, diabetes requiring insulin, and preoperative serum creatinine >2 mg/dL [17]. For each risk factor, 1 point is assigned. Patients with 0, 1, 2, and, finally, 3 or more factors are assigned to classes I, II, III, and IV, respectively. Cardiac complications increase from lower to higher classes [18] (Table 1).

The role of echocardiography, stress imaging for coronary artery disease, and, finally, coronary CT or coronary angiography is still a matter of debate. In patients who need emergent/urgent surgery, a bedside echocardiography may add significant elements to the history and ECG for risk stratification, particularly in elderly patients, in whom functional capacity and symptoms may not be valuable due to limited physical activity, or with left-sided murmurs. Left ventricle wall motion abnormalities, left and right ventricular function, hemodynamic-relevant valve disease, pulmonary artery pressure estimates, and, finally, evaluation of overall volume status may give prognostic information, guide volume replacement, and suggest the need for hemodynamic monitoring to limit postoperative complications. Echocardiography does not take more than a few minutes and skilled cardiologists or anesthesiologists should be included in the group for preoperative evaluation. ESC 2022 guidelines [5] suggest echocardiography in class 1 B for patients with poor functional capacity and/or high NT-pro-BNP BNP, or if murmurs are detected before high-risk NCS while TTE should be considered in patients with suspected new CVD or unexplained signs or symptoms before high-risk NCS (IIa, B recommendation). In patients who need elective surgery, a comprehensive evaluation should be considered for the identification of CAD and its severity. Symptomatic patients should be assessed and treated according to guidelines and surgery delayed.

In asymptomatic patients, stress imaging is recommended before high-risk elective NCS in subjects with poor functional capacity and a high likelihood of CAD or high clinical risk (evidence Ia). Stress imaging should be considered before high-risk NCS in asymptomatic patients with poor functional capacity, and previous PCI or CABG (Evidence IIb). Further management requires the multidisciplinary assessment and evaluation of the risk–benefit ratio between CAD management and scheduled surgery.

## 4. Pathogenesis

PMI after non-cardiac surgery has different underlying pathogenetic mechanisms. According to the fourth universal definition of infarction, most MINS may be classified as type 2 myocardial infarctions.

Type 1 myocardial infarction is due to acute plaque rupture, ulceration leading to occlusive coronary thrombosis, and severe acute myocardial ischemia. It is usually associated with ST elevation at ECG. In the postoperative period, this condition is infrequent (not more than 5–10%). The postoperative inflammatory state may have a pivotal role in the pathogenesis of type 1 MI. Inflammation acts as a trigger, exacerbating ischemic heart damage, embolic vessel obstruction, and thrombosis. The OPTIMUS study [19] included 30 patients with perioperative MI and 30 patients with non-perioperative MI who were studied with CT. Thrombosis was found in the culprit vessel in only 4 out of 30 patients (13%) with perioperative MI, in comparison to 20 out of 30 (66.7%) who had had spontaneous MI. Nevertheless, patients with MINS frequently had coronary artery disease and fibroatheroma was demonstrated in 18 patients (60%) with perioperative MI.

Type 2 postoperative myocardial infarction is the consequence of a mismatch between myocardial oxygen demand and supply in the absence of coronary thrombosis. This occurs more frequently in patients with known or occult coronary artery disease in whom acute stressors may result in myocardial ischemia and injury reflected by troponin release. In the postoperative setting, patients rarely suffer from chest pain, while dyspnea or unappropriated tachycardia may raise clinical suspicion, and the ECG gives information in less than 50% of patients; therefore, diagnosis often depends on a troponin assay (Table 2). Surgical stress, bleeding, hypotension related to hypovolemia or altered vasoreactivity, the activation of hemostasis, and a marked inflammatory response (CRP, TNFα, IL-1, and IL-6) can lead to reduced oxygen supply to the myocardium. The same systemic inflammation, resulting from surgical trauma, increases the myocardial oxygen demand. After The presence of tachycardia also increases the oxygen demand, limits the perfusion time during diastole, and contributes to myocardial damage [15].

The occurrence of tachyarrhythmias, acute heart failure (AHF), and extracardiac situations such as severe sepsis and pulmonary embolism are not uncommon causes of hs TnT increase after surgery.

The two types of PMI have different incidences depending on the type of surgery: type 2 perioperative infarctions often follow general, orthopedic, and thoracic surgery, while type 1 infarctions are more frequently associated with vascular surgery.

## 5. Clinical Manifestations

Classic symptoms of myocardial ischemia are infrequent in patients with MINS. Chest pain is often masked by the administration of analgesic drugs for postoperative pain management, or by anesthesia itself or postoperative sedation. Dyspnea may be frequently overlooked as a symptom of ischemia. More than 65% of patients with PMI are asymptomatic, ECG monitoring is rarely applied, and this accounts for the low detection rate before the introduction of biomarkers. Due to variable ECG monitoring, ECG signs of myocardial ischemia are often missed. For example, in the international POISE-1 trial among 415 patients who had a perioperative myocardial infarction, approximately 65 percent were asymptomatic [20]. Only 2% of patients had ST elevation at ECG.

## 6. Screening

The use of troponin I or T and, more recently, of high-sensitivity troponin I and T (hs-cTnI, hs-cTnT) allowed for diagnosing postoperative myocardial damage/infarction, which is otherwise frequently missed [21,22]. Potentially confounding factors may be the type of method used for the troponin assay, the presence of preoperative values already outside the reference range (above the 99th upper reference limit), and the presence of chronic release-reduced elimination conditions (heart failure, renal failure).

The adoption of the hs-cTn assay increased the sensitivity of the method but has significantly reduced the specificity, often creating interpretative difficulties that are not easy to solve. The increase in preoperative values must be contextualized in the clinical situation: in the patient who requires emergency time-dependent surgery, the underlying clinical problem may have triggered hemodynamic, inflammatory, and metabolic alterations capable of causing myocardial damage prior to and independent from the intervention. The possible interpretation of elective surgery is quite different.

Few studies have compared different troponin assay methods. In a prospective study recently concluded and under evaluation, our group compared cTnI with hs-cTnT assays in patients undergoing hip fracture surgery. An increase above the 99th percentile of the URL was found in 92.5% of patients when hs-cTnT values were assayed in comparison to 29% when cTnI was assayed. In-hospital and 1-year mortality of patients with a peak troponin level <99th of the URL was, respectively, 0 and 2% for hs-CTnT and 4.1 and 23% for cTNI. For values >99th percentile of the URL, an incremental risk of both in-hospital and 1-year mortality was observed with both methods, as well as an increase in hospitalization for cardiac causes. Pelaucher et al. [23] compared hs-cTnI and hs-cTnT. The incidence of overall PMI was 9% lower using hs-cTnT (overall PMI 15%). The difference has been attributed to a non-biological equivalence of approved URLs, but a different release pattern after perioperative triggers might contribute as well.

Independently from the assay used, a preoperative troponin value may allow for differentiating acute from chronic increases. The preoperative measurement of troponin has sometimes been questioned since patients with high values (perhaps even chronic) may see their surgery unnecessarily delayed. Otherwise, acute clinical conditions, for example, hip fracture, may be associated with high preoperative troponin values that are associated with a worse prognosis [14,24].

In the case of a high postoperative cTn concentration in the absence of a prior cTn measurement (pre- or postoperative), a second cTn measurement should be obtained to determine whether a rising or falling pattern exists suggesting acute myocardial damage [25].

Elevated troponin values are not synonymous with MINS; a “chronic” increase in blood troponin has been reported in several investigations and may be due to non-ischemic causes, which should be excluded. In the VISION study, among patients with high postoperative hs-cTn levels, 13.8% had preoperative values greater than or like the postoperative peak.

Patients with MINS should be evaluated for cardiovascular risk factors, treated for any contributing conditions (e.g., anemia), considered for medical therapy, and appropriately evaluated for obstructive CAD (e.g., stress echocardiography, stress radionuclide myocardial perfusion, coronary computed tomographic angiography).

The European Society of Cardiology issued a Class IB recommendation for active surveillance for PMI; a preoperative 12-lead ECG is recommended in all intermediate- and high-risk patients with symptoms of ischemic heart disease, cardiovascular disease, and cardiovascular risk [5]. Preoperative BNP or NT-proBNP dosing should be considered. Screening for MINS with a measurement of troponin blood levels and ECG perioperatively is indicated in high-risk patients according to the RCRI [5]. Surveillance with high-sensitivity cTn should extend to 24 and 48 h after surgery. Similar recommendations were made by the American Heart Association [26]. The recently released ESAIC-focused guideline for the use of cardiac biomarkers in perioperative risk evaluation [27] more accurately deals with the timing (preoperative, postoperative, and pre- and postoperative) of troponin measurement in patients undergoing non-cardiac surgery. For each strategy, the prognostic value, the predictive capacity of postoperative events, and, finally, the role in guiding the postoperative management of complications were considered. None of these strategies has more than a weak grade of recommendation; however, the use of postoperative cardiac troponins for prognosis and risk prediction was compared to serial pre- and postoperative values (increased postoperative over preoperative values). These studies generally investigated non-high-sensitivity Tn assays. In light of these differences, larger prospective studies with clearly definite end points are needed to guide clinicians. The ESAIC guidelines suggest that the preoperative measurement of NT-pro-BNP should be considered to improve the risk stratification of adverse events, in particular, 30-day MACE, 30-day death, and myocardial infarction, to inform patients and encourage shared decision-making. In the postoperative period, at present, the measurement of NT-pro-BNP should only be considered for research purposes.

## 7. Diagnosis

In patients who present with typical signs or symptoms of myocardial infarction after surgery, there are no differences from those who present with signs or symptoms of myocardial infarction in other settings. Differential diagnosis should be considered for patients with nonspecific signs and troponin increase, in particular, pulmonary embolism. In patients who underwent postoperative screening and have an elevated troponin level, an ECG and troponin assay should be repeated and compared with previous examinations and any preoperative data available. Since the ECG has a low sensitivity, the cardiac troponin measurement is critical to detect PMI. The diagnosis is made when there is an absolute increase in the blood concentrations of cardiac troponins, cTn, and high-sensitivity cT (hs-cTnT and hs-cTnI), above the 99th percentile associated with typical symptoms of myocardial ischemia and pathognomonic changes in instrumental examinations [28]. Among patients with elevated troponin levels, only a small number have electrocardiographic abnormalities. In the VISION study, in which a non-high-sensitivity troponin was dosed, approximately 35% of patients had altered electrocardiographic findings [8]. A significant number of patients with MINS likely have electrocardiographic abnormalities at some point in the postoperative course. But these changes are rarely appreciated: several patients do not perceive typical symptoms that require an ECG during ischemia; most ECGs are performed after detecting the presence of an elevated troponin level, which is usually observed about 24 h after surgery, and do not show ischemic abnormalities; the increase in troponin levels usually occurs after the ischemic event has already begun. Therefore, the differential diagnosis between PMI and MINS may be often missed.

When the diagnosis remains uncertain, additional noninvasive studies (e.g., echocardiography, radionuclide myocardial perfusion imaging, cardiovascular magnetic resonance imaging) may be required to confirm or exclude the presence of myocardial infarction. The approach to additional testing in patients who recently underwent surgery should be like that in patients in the general population; however, the availability of examination may be limited, and the patient’s condition as well may delay the study. 

In patients diagnosed with myocardial injury after non-cardiac surgery, it is reasonable after recovery to obtain stress or anatomic imaging to assess for obstructive CAD unless another clear explanation for an elevated troponin level is present. However, the small number of prospective studies that describe the yield of such testing do not allow us to draw conclusions.

## 8. Treatment

Preoperative treatment with antiplatelets, statins, beta blockers, and ACEi in high-risk patients is associated with a decreased risk of PMI and overall better outcomes. According to updated ESC guidelines, patients with indications for statins should start taking statins perioperatively, and it is recommended to continue taking statins and beta blockers perioperatively if they were previously taken. Perioperative troponin monitoring after non-cardiac surgery showed that the 30-day myocardial infarction and cardiovascular mortality rate were reduced by 25% after the initiation of treatment with acetylsalicylic acid and statins in high-risk patients.

In patients without heart failure, the discontinuation of RAAS (renin–angiotensin system) inhibitors should be considered on the day of surgery to prevent perioperative hypotension, while those with stable heart failure could continue to take RAAS inhibitors. 

In the small cohort of patients with perioperative STEMI, the approach to management is like that in other patients with STEMI, provided that the risk of bleeding with antiplatelet agents and anticoagulation may be reasonable. In patients with a high bleeding risk, the use of angiography and percutaneous coronary intervention (PCI) must be individualized.

Apart from ST-elevated myocardial infarction, the high 1-year mortality associated not only with PMI but also with MINS raises several questions about postoperative management. In patients with myocardial injury, it has been suggested to reevaluate the patient two weeks after discharge to manage cardiovascular risk factors, monitor therapy, and eventually determine the need for imaging to detect obstructive CAD. Anatomic imaging of the coronary arteries (CT) to assess for CAD should be obtained in patients with MINS and without significant cognitive or functional impairment. The advantage of such a strategy is not supported by randomized studies. As well, available data are still insufficient to support the usefulness of coronary angiography. A study including 34,650 patients with perioperative MI showed that coronary angiography was associated with lower in-hospital mortality compared to medical treatments (8.9 vs. 18.1%) [29]. The presence of major bleeding due to double antiplatelet therapy is associated with a worse outcome after non-cardiac surgery.

In a study by Parashar et al. [30] from 2003 to 2012, a total of 1093 patients underwent diagnostic coronary angiography; 281 underwent PCI. The mortality rate after perioperative myocardial infarction remained high even after PCI: one-year mortality was 15% in the overall population. In the PCI subpopulation, mortality was 11.3% with more than 1/4 patients and 1/10 patients not surviving beyond 30 days after STEMI and NSTEMI, respectively. Risk analysis showed that increasing age, bleeding after PCI, renal failure, and vascular surgery are all significant predictors of long-term mortality after PCI. In a small group of patients with postoperative MI after hip surgery, we found a significant decrease in mortality after revascularization in comparison to age and sex-matched patients who did not undergo angiography [31]. Similar results were reported in another study [32]. In clinical practice, however, the postoperative evaluation of patients with MINS is still largely overlooked.

About medical treatment, in patients without a significant risk of bleeding, the use of dabigatran for two years after MINS may be considered, based on the MANAGE study in which 110 mg twice daily of dabigatran was administered [33]. The use of dabigatran in patients who developed MINS (started on average 7 days after surgery) has been shown to be effective in reducing the risk of major cardiovascular events by 30% (Hazard Ratio = 0.7; 95% confidence interval: 0.55–0.93), and without increasing the risk of postoperative bleeding. In an observational sub-study of POISE-1, aspirin and statin use were each associated with a reduction in the risk of 30-day mortality among patients who had suffered a perioperative myocardial infarction (adjusted OR for aspirin 0.54, 95% CI 0.29–0.99 and adjusted OR for statins 0.26, 95% CI 0.13–0.54) [34].

## 9. Prognosis

The presence of MINS is associated with a worse prognosis in the short-, medium-, and long-term. MINS that do not meet the universal diagnosis of perioperative myocardial infarction are independently associated with mortality after 30 days. Long-term mortality is increased in patients with elevated troponin blood levels, regardless of whether there is a diagnosis of PMI or MINS. An incremental risk is associated with higher peak troponin values [4]. A prospective study showed that 30-day mortality was 8.9% in patients with MINS and 1.5% in comparison to the controls [35]. In addition, there was no difference in 30-day mortality between those who had true MI and those who did not meet the criteria and were diagnosed with MINS. Long-term mortality (at one year) was also higher in patients with MINS (22.5%) than in those without (9.3%).

Perioperative MI is associated with an increased risk of death at 30 days. Although all etiologies of PMI are associated with an increased risk of death within 1 year, the mortality rate differs substantially between causes. A large prospective international multicenter study showed that mortality and the development of MACE within one year increased in patients with PMI due to heart failure and tachyarrhythmia [36]. Similar risks are present in patients with type 1 MI. Patients who develop PMI due to a mismatch between oxygen supply and demand have lower mortality.

Nonfatal perioperative MI is a major risk factor for acute coronary syndrome, nonfatal cardiac arrest, heart failure, stroke, 30-day rehospitalization, or progressive angina requiring revascularization after surgery [7]. Patients with PMI have an increased risk of readmission to the hospital within 90 days compared to patients without PMI. No major differences are observed between the various underlying causes except that they are more likely to be readmitted to type 1 SMEs than other etiologies [37].

A large U.S. study identified 8085 patients who were diagnosed with perioperative MI [35]. These patients, compared to those without MI, have a 13% higher absolute mortality and a longer hospital stay of six days. Among surviving patients, the rehospitalization rate was higher in patients with MI (19.1%) than in those without MI (6.5%). The main causes of rehospitalization at 30 days were infectious complications (30%), cardiovascular complications (25.3%), and hemorrhagic complications (10.4%) among patients with MI; infectious (30.6%), gastrointestinal (12.0%), and cardiovascular (8.2%) complications among patients without MI.

Regarding in-hospital mortality, depending on the type of surgery in patients with perioperative MI, a study conducted by Smilowitz et al. [35] showed that the surgeries most at risk are thoracic surgery (28.4%), general surgery (15.3%), and neurosurgery (12.5%) (Table 3).

In the VISION study [8], 30-day mortality was 1.2% (266 patients). In this study, an increase in high-sensitivity troponin values >5 ng/L between two different measurements without ischemic symptoms was associated with an increase in 30-day mortality. In addition, an increase in maximum values had prognostic relevance: patients with hs-cTn 20–65 ng/L had a 30-day mortality of 3%, with hs-cTn 65–1000 ng/L of 9.1%, and with hs-cTn > 1000 ng/L of 29.6%.

## Figures and Tables

**Table 1:** MACE according to Revised Cardiac Risk Index.

Risk Class	30-Day Risk of Death, MI, or Cardiac Arrest
0	0.6%
1	0.9%
2	6.6%
3 or more	11%

**Table 2:** Symptoms and ECG changes in postoperative myocardial infarction/injury.

	PMI 397	Cardiac PMI 342	Non-Cardiac PMI 55
Chest pain	24–6%	19–6%	5–9%
Dyspnea	46–12%	39–11%	7–13%
Any possible ischemia-related symptom	72–18%	59–17%	13–24%
ECG changes	60–24%	54–25%	6–23%

Modified from [20].

**Table 3:** Mortality and 30-day rehospitalization in function of type of surgery.

Type of Surgery	Death (%)	30-Day Rehospitalization (%)
Thoracic	28	12
General	20	15
Vascular	15	16
Orthopedic	11	17
Skin/breast	7	21
Genitourinary	7	20
Otolaryngology	5	18
Neurosurgery	13	8

## Data Availability

Not applicable.
